# Three-dimensional computed tomography image-oriented successful thoracoscopic subtotal esophagectomy for an esophageal cancer patient with an anomalous right superior pulmonary vein: A case report

**DOI:** 10.1016/j.ijscr.2020.09.196

**Published:** 2020-10-02

**Authors:** Takeshi Matsubara, Noriyuki Hirahara, Hitomi Zotani, Nariyasu Tabara, Hideki Tabara, Yoshitsugu Tajima

**Affiliations:** a Department of Surgery, Izumo Tokushukai Hospital, Japan; b Department of Digestive and General Surgery, Shimane University Faculty of Medicine, Japan

**Keywords:** RSPV, right superior pulmonary vein, 3D-CT, three-dimensional contrast-enhanced computed tomography, VATS-E, video-assisted thoracoscopic surgery-esophagectomy, SCC, squamous cell carcinoma, MDCT, multidetector-row computed tomography, 3D-CT, Right superior pulmonary vein, Aberrant V2, Subcarinal lymphadenectomy, Esophageal cancer

## Abstract

**Background:**

Subcarinal lymphadenectomy is an essential procedure in curative esophagectomy for esophageal cancer. The right superior pulmonary vein (RSPV) and its branches are usually located in front of the right main or intermediate bronchus. However, an anomalous posterior branch (aberrant V2) of RSPV passes behind the right intermediate bronchus, where the aberrant V2 may be embedded in the subcarinal nodal packet. This can lead to unanticipated bleeding when dissecting the subcarinal lymph node. We present a case study on the use of preoperative three-dimensional contrast-enhanced computed tomography (3D-CT) for performing a safe video-assisted thoracoscopic surgery-esophagectomy in lower thoracic esophageal cancer.

**Case presentation:**

A 77-year-old man had esophageal cancer associated with an aberrant V2 passing behind the right intermediate bronchus. Esophagogastroduodenoscopy revealed a type 1 tumor in the lower thoracic esophagus. Contrast-enhanced and 3D-CT scans showed a space-occupying lesion with contrast enhancement and an aberrant V2 passing behind the right intermediate bronchus, respectively. The patient was then diagnosed with lower thoracic esophageal cancer (cT2cN1cM0 cStage II). As per the patient’s request, he underwent a surgery-first approach followed by adjuvant chemotherapy. The patient underwent video-assisted thoracoscopic surgery-esophagectomy (VATS-E) with three-field lymphadenectomy, and a large aberrant V2 involving the subcarinal nodal packet was recognized behind the right intermediate bronchus. After the thoracoscopic subtotal esophagectomy with three-field lymph node dissection, laparoscopy-assisted reconstruction of the esophagus was performed with elevation of the gastric conduit to the neck. Recurrent laryngeal nerve palsy was not observed. He started to receive rehabilitation for swallowing on day 3 and resumed oral intake on day 10 after surgery. The final pathological diagnosis was squamous cell carcinoma of the esophagus (pT3N1M0 pStageIII).

**Conclusions:**

Preoperative contrast-enhanced 3D-CT clearly depicted the aberrant V2, which enabled us to perform a safe VATS-E with three-field lymphadenectomy. Thorough understanding of the anatomical configuration of the pulmonary vessels and bronchus is important for avoiding unexpected bleeding during subcarinal lymphadenectomy. 3D-CT imaging study is useful for recognizing the anomalous RSPV before surgery.

## Highlights

• Case of esophageal cancer associated with an aberrant V2.
• Used preoperative 3D-CT for performing safe VATS-E.
• Preoperative contrast-enhanced 3D-CT clearly depicted the aberrant V2.

## 1 Background

Esophagectomy with three-field lymphadenectomy is the gold standard surgical procedure for thoracic esophageal cancer in Japan. To achieve curative resection, subcarinal lymphadenectomy is one of the essential processes [1].

In most cases, branches of the right superior pulmonary vein (RSPV) are located inside the right lung and run in front of the right main or intermediate bronchus. However, these vessels rarely pass behind the right bronchus. In such patients, identifying aberrant vessels is quite important to avoid unanticipated bleeding, especially when dissecting the subcarinal lymph node. This is because even a slight bleeding in this space may lead to disorientation of an adequate dissection layer or surgical anatomy, resulting in intraoperative serious complications. Accordingly, thorough understanding of the anatomical configuration of the pulmonary vessels and bronchus is essential for the prevention of intraoperative complications in thoracic surgery, including esophagectomy.

Herein, we report a case of lower thoracic esophageal cancer with an anomalous posterior branch (aberrant V2) of RSPV passing behind the right intermediate bronchus, wherein preoperative three-dimensional contrast-enhanced computed tomography (3D-CT) provided useful information for performing a safe video-assisted thoracoscopic surgery-esophagectomy (VATS-E) with three-field lymphadenectomy, including the subcarinal lymph nodes.

## 2 Case presentation

**Fig. 1:**
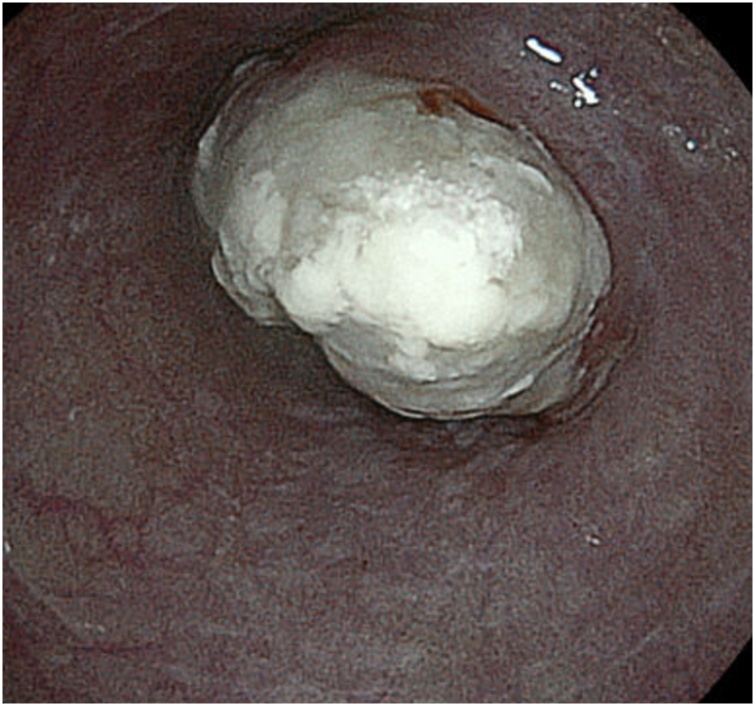
Upper gastrointestinal endoscopy imaging. The upper gastrointestinal endoscopic examination shows a protruding tumor in the lower thoracic esophagus.

**Fig. 2:**
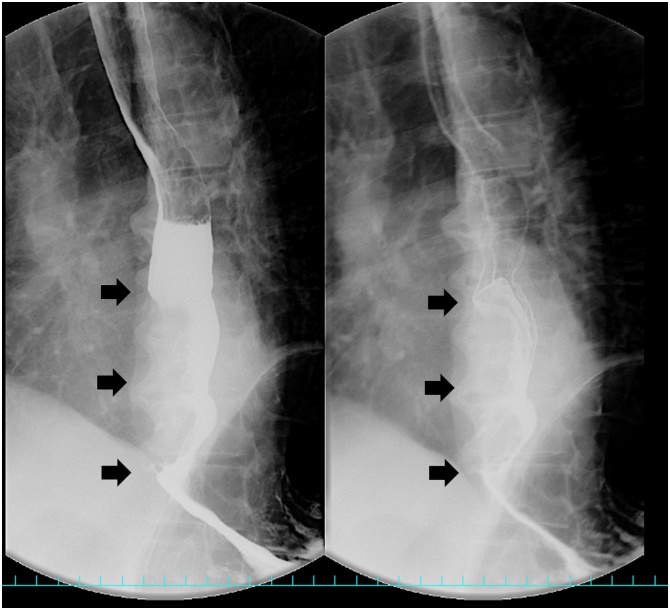
Barium esophagography scan. The scan reveals a space-occupying lesion in the lower thoracic esophagus (arrows).

**Fig. 3:**
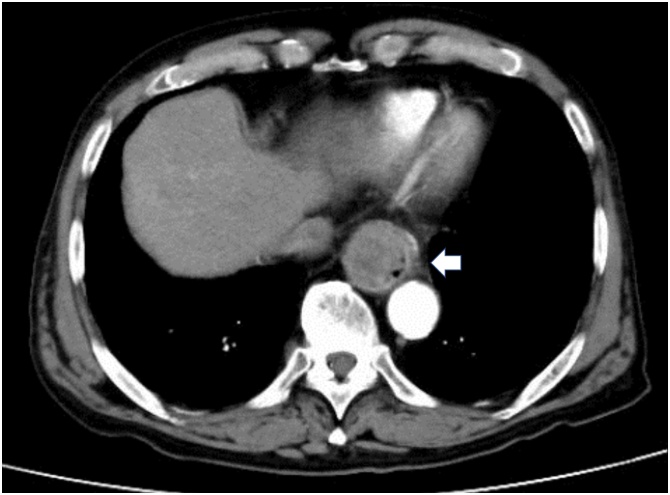
Contrast-enhanced computed tomography (CT) scans. The scan shows a 6-cm-long mass lesion with contrast enhancement in the lower thoracic esophagus (arrow).

**Fig. 4:**
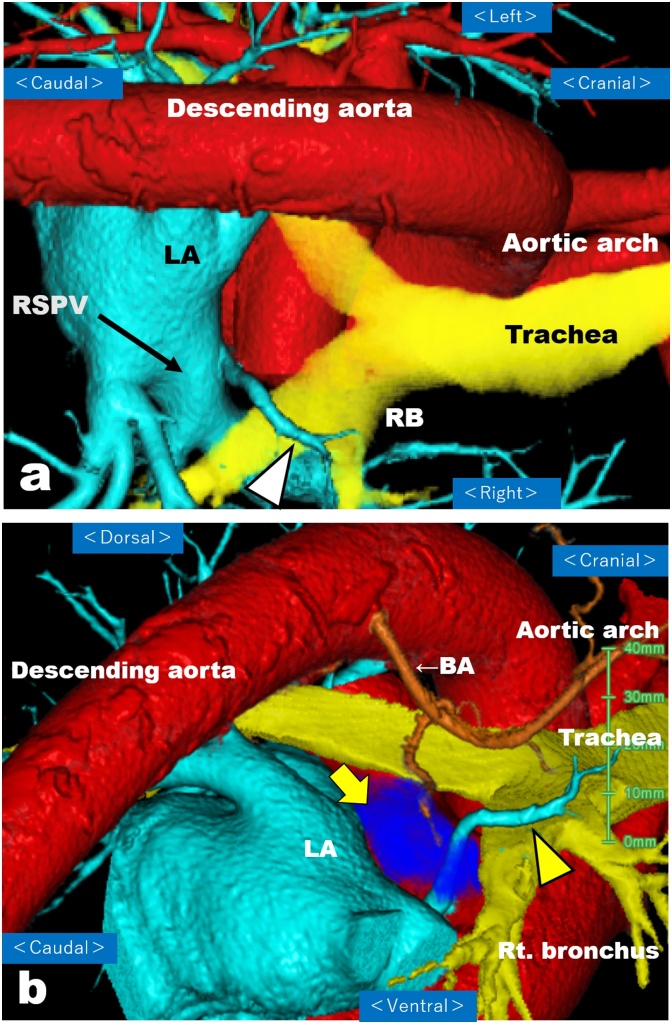
(a) Preoperative reconstructed three-dimensional computed tomography (3D-CT) imaging. The image shows an aberrant V2 (arrowhead) passing behind the right intermediate bronchus and merging with RSPV (arrow). LA: left atrium, RSPV: right superior pulmonary vein, RB: right bronchus. (b) Preoperative reconstructed three-dimensional computed tomography (3D-CT) imaging. The image reveals the aberrant V2 (arrowhead) and branches of the right bronchial artery (thin arrow) running through the subcarinal nodal packet (thick arrow). BA: right bronchial artery, LA: left atrium.

A 77-year-old man presented with swallowing difficulty and 10-kg weight loss for the past 3 months and therefore was referred to our hospital. Past medical history included hypertension and bilateral inguinal hernias. The patient’s height and weight were 175 cm and 58 kg, respectively, with a body mass index of 18.9 kg/m^2^ and performance status of 0. Preoperative blood tests revealed low levels of serum albumin (3.5 g/dl) and a slight elevation of squamous cell carcinoma (SCC) antigen (4.9 ng/mL). Esophagogastroduodenoscopy (Fig. 1) showed a type 1 tumor in the lower thoracic esophagus, and pathological analysis of the tumor biopsy demonstrated SCC. Barium esophagography (Fig. 2) showed a bulging lesion, 6-cm long, in the lower esophagus with mild dilatation of the upper esophagus. A complete esophageal obstruction was not observed. Contrast-enhanced multidetector-row computed tomography (MDCT) scans showed a space-occupying lesion with contrast enhancement, measuring 6 × 3 cm, in the lower thoracic esophagus (Fig. 3). An enlarged lymph node, 13 mm in size, was observed along the left gastric artery. Moreover, 3D-CT images clearly depicted an aberrant V2 passing behind the right intermediate bronchus (Fig. 4a), penetrating the subcarinal nodal packet (Fig. 4b), and merging with RSPV (Fig. 4a). The 3D-CT images were obtained using the Aquilion Prime SP CT Scanner (Canon Medical Systems Corporation, Tochigi, Japan) and were reconstructed using a 3D workstation (Ziostation2®, Ziosoft Inc., Tokyo, Japan). Distant metastases were not observed.

The patient was finally diagnosed with lower thoracic esophageal cancer (cT2cN1cM0 cStage II) according to the Japanese Classification of Esophageal Cancer [2]. Although neoadjuvant chemotherapy is strongly recommended for esophageal cancer patients with lymph node metastasis [3], the patient wanted to undergo a surgery-first approach followed by adjuvant chemotherapy with prior informed consent.

**Fig. 5:**
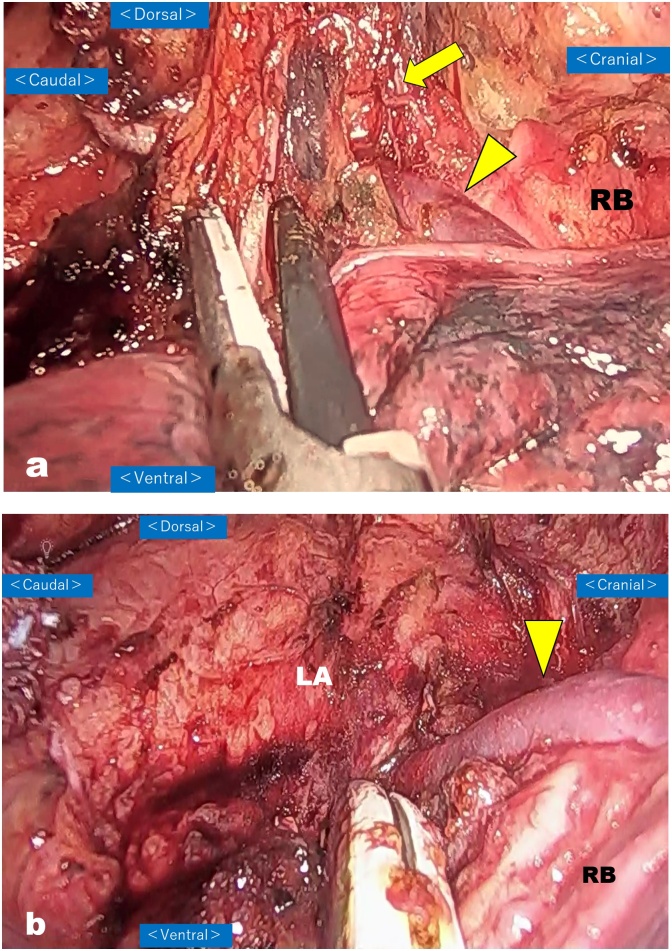
(a) Subcarinal lymph node dissection. View of the subcarinal lymph node dissection showing the aberrant V2 (arrowhead) embedded in the subcarinal nodal packet (arrow). RB: right intermediate bronchus. (b) Subcarinal lymph node dissection. View of the subcarinal lymph node dissection showing the completion of subcarinal lymphadenectomy without injury to the aberrant V2 (arrow). LA: left atrium, RB: right intermediate bronchus.

The patient underwent VATS-E with three-field lymphadenectomy using 4 ports under artificial CO_2_ pneumothorax (CO_2_ pressure: 6–10 mmHg) in the prone position. As depicted on the preoperative 3D-CT images, a relatively large aberrant V2 involving the subcarinal nodal packet was recognized behind the right intermediate bronchus (Fig. 5a). When dissecting the subcarinal lymph nodes, we usually expose the surface of the pericardial membrane in the caudal-to-cranial direction to reach the caudal aspect of the right bronchus in the first step. In this patient, we exposed the pericardium up to the lower bulge of RSPV, i.e., near the area where the aberrant V2 merged with RSPV, and then switched the dissection plane to the membranous portion of the right bronchus. After that, the aberrant V2 was carefully exposed from the distal side. The dissection continued along the aberrant V2 to the proximal direction and proceeded ventrally. After reaching near the root of RSPV, the nodal dissection on the pericardium was advanced cranially, and subcarinal lymphadenectomy was completed without injury to this anomalous vein (Fig. 5b).

After completing thoracoscopic subtotal esophagectomy with three-field lymph node dissection, laparoscopy-assisted reconstruction of the esophagus was performed with elevation of the gastric conduit to the neck through the posterior mediastinal approach. The operation time was 620 min, and the estimated intraoperative blood loss was 150 mL.

The postoperative course was uneventful. The next day after surgery, the patient was extubated. Recurrent laryngeal nerve palsy was not observed. He started to receive rehabilitation for swallowing on the third day and resumed oral intake on the tenth day after surgery. The final pathological diagnosis was squamous cell carcinoma of the esophagus, pT3N1M0 pStageIII, according to the Japanese Classification of Esophageal Cancer [2].　Total number of dissected lymph nodes was 38, and the subcarinal group of lymph nodes specifically was 7. There was no subcarinal lymph node metastasis. The work has been reported in line with the SCARE 2018 criteria [4].

## 3 Discussion and conclusions

The venous drainage system from the right upper lobe of the lung usually composes of three main segmental veins, namely, apical branch (V1), posterior branch (V2), and anterior branch (V3), which ordinally run inside the right lung and merge with RSPV. Although left-sided aberrant pulmonary venous return is uncommon, some anomalies of the right pulmonary vein such as aberrant V2, V4, and V6 have been reported as highly frequent among surgical cases of lung cancer surgery [5,6]. In such cases with aberrant V2, there could be pitfalls including massive bleeding during subcarinal lymphadenectomy because anomalous RSPV is likely to involve the subcarinal nodal packet.

The incidence of aberrant V2 has been reported to be 2.2%–5.7% [[7], [8], [9], [10]]. Arslan et al. [6] detected aberrant V2 using MDCT in 2.2% of 610 patients with suspected thoracic or cardiac pathology. Akiba et al. [11] evaluated the frequency and anatomical configuration of anomalous V2 using 3D-CT images in 303 patients with chest disorders. They found aberrant V2 in 10 patients (3.3%) and categorized these anomalies into six types based on their route and inflow site. The aberrant V2 passed behind the right intermediate bronchus in 8 cases, passed behind the right main bronchus in one, and crossed behind both the right main and intermediate bronchi in one. The inflow site was the left atrium in 4 cases, RSPV in 4, right inferior pulmonary vein in one, and V6 in one. The aberrant V2 in our patient, passing behind the right intermediate bronchus and merging with RSPV, is the most common type according to the classification of Akiba et al. [11].

Only 4 case reports describing the association of anomalous RSPV and subcarinal lymphadenectomy for esophageal cancer are available in literature [[12], [13], [14], [15]]. Matsubara et al. [12] reported a case of aberrant V2 originating from the right upper lobe and passing behind the right intermediate bronchus with penetration of the subcarinal nodal packet. They mentioned that they encountered 2 such cases with aberrant V2 (0.28%) in a series of 700 esophagectomies. Fujiwara et al. [13] reported an aberrant V2 passing behind the right intermediate bronchus and draining into the left atrium, which was recognized during subcarinal lymphadenectomy in thoracoscopic esophagectomy for esophageal cancer. Shiozaki et al. [14] reported an aberrant V2 identified during laparoscopic transhiatal lower esophagectomy for esophageal cancer. In that case, the aberrant V2 passed behind the right intermediate bronchus, penetrated the subcarinal nodal packet, and drained into the left atrium. In addition, these 3 aberrant V2 were identified during esophagectomy (intraoperatively) and were reconfirmed postoperatively by reviewing preoperative contrast-enhanced CT images. On the other hand, one case was reported in which a preoperative diagnosis by imaging was possible. Onodera et al. [15] described an aberrant V2 in a patient with lower esophageal cancer, which was depicted on preoperative 3D-CT scans to pass behind the right intermediate bronchus and drain into RSPV. As mentioned above, an aberrant V2 can be identified by using contrast-enhanced CT and an exact preoperative 3D-anatomical recognition of anomalous RSPV is important to reduce the risk of fatal complications during subcarinal lymphadenectomy.

A precise depiction of aberrant V2 on preoperative reconstructed 3D-CT images enabled us to make up a strategic plan for safely performing esophagectomy with subcarinal lymphadenectomy in our patient. If the aberrant V2 was injured, it might have resulted in serious bleeding under circumstances of a narrow surgical space. An aberrant V6 flowing into the right inferior pulmonary vein or left atrium has also been reported [8]. VATS-E with artificial pneumothorax in the prone position may provide a good surgical field of view for identifying anomalous RSPV because the posterior upper lobe segment (S2) is anatomically located in the dorsal part of the right upper lobe of the lung. In this instance, an aberrant V6 could be recognizable during VATS-E with the patient in the prone position. Thus, prone VATS-E would be beneficial for esophageal cancer patients with an aberrant pulmonary vein under preoperative 3D-CT image guidance.

In summary, an aberrant V2 was clearly depicted on preoperative contrast-enhanced 3D-CT in a patient with lower thoracic esophageal cancer, which aided in a safe VATS-E with three-field lymphadenectomy, including subcarinal lymph nodes. Thorough understanding of anatomical configuration of the pulmonary vessels and bronchus is important for avoiding unexpected bleeding during subcarinal lymphadenectomy. 3D-CT imaging is useful for recognizing the anomalous RSPV before surgery.

## Declaration of Competing Interest

The authors report no declarations of interest.

## Funding

The authors declare that they have no competing interests and received no financial support.

## Ethical approval

All procedures followed were in accordance with the Ethical Guidelines for Medical and Health Research Involving Human Subjects in Japan and with the Helsinki Declaration of 1964 and later versions.

## Consent

Written informed consent was obtained from the patient for publication of this case report and accompanying images. A copy of the written consent is available for review by the Editor-in-Chief of this journal on request.

## Authors contribution

TM, HN, HZ and NT were operator and assistant for this patient. HT and YT were major contributor in writing the manuscript. All authors read and approved the final manuscript. HZ and NT conducted the literature search and drafted the manuscript. TM and NH contributed to the conception and design of the work. TM,　HN, HZ　and NT were involved in the management of the patient. HT and YT were major contributor in writing the manuscript. All authors reviewed the manuscript and gave approval for publication of the final version.

## Registration of research studies

Our case report is not a first-in-man study.

## Guarantor

Takeshi Matsubara.

## Provenance and peer review

Not commissioned, externally peer-reviewed.
